# Endometriosis in Adolescence: A Narrative Review of the Psychological and Clinical Implications

**DOI:** 10.3390/diagnostics15050548

**Published:** 2025-02-24

**Authors:** Fabiola Panvino, Roberto Paparella, Francesco Pisani, Francesca Tarani, Giampiero Ferraguti, Marco Fiore, Ignazio Ardizzone, Luigi Tarani

**Affiliations:** 1 Department of Human Neuroscience, Sapienza University of Rome, 00185 Rome, Italy; fabiola.panvino@uniroma1.it (F.P.);; 2 Department of Maternal Infantile and Urological Sciences, Sapienza University of Rome, 00185 Rome, Italy; roberto.paparella@uniroma1.it (R.P.);; 3 Department of Experimental Medicine, Sapienza University of Rome, 00185 Rome, Italy; 4 Institute of Biochemistry and Cell Biology (IBBC-CNR), Department of Sensory Organs, Sapienza University of Rome, 00185 Rome, Italy

**Keywords:** endometriosis, chronic pelvic pain, well-being, adolescence, anxiety, depression

## Abstract

Endometriosis is a chronic, inflammatory condition where endometrial-like tissue grows outside the uterus, affecting around 10% of women of reproductive age. This condition is associated with debilitating symptoms, including dysmenorrhea, dyspareunia, chronic pelvic pain, fatigue, and infertility. Adolescents with endometriosis face unique challenges, as the disease is often misdiagnosed or undiagnosed for an average of 7–10 years due to its complex and multifactorial nature. Consequently, patients frequently suffer from worsening symptoms and significant psychological distress, including anxiety, depression, and social withdrawal. While there is no definitive cure for endometriosis, treatment approaches typically involve hormonal therapies, lifestyle adjustments (such as diet and exercise), and psychological support. Recent studies emphasize the profound impact of endometriosis on the mental health of adolescents, highlighting the need for a more holistic treatment approach that integrates both medical and psychological care. This narrative review explores the psychological and psychosocial effects of endometriosis in adolescents, examining the biological and psychological mechanisms linking the disease to mental health outcomes. It also discusses current therapeutic strategies, such as cognitive behavioral therapy, mindfulness, and peer support, and underscores the importance of early diagnosis and multidisciplinary care to mitigate both the physical and emotional burdens of the condition. This integrated approach is critical in improving the overall well-being and quality of life for adolescents living with endometriosis.

## 1. Introduction

**Figure 1:**
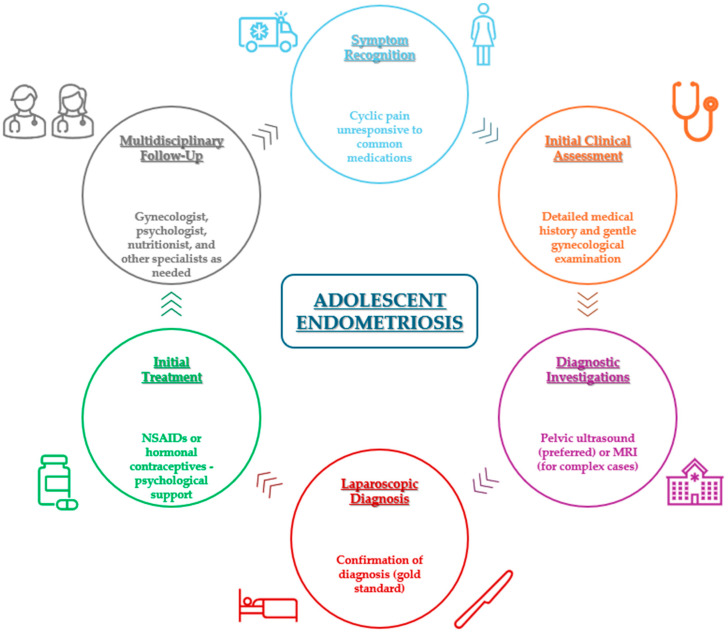
A visual representation of the classical diagnostic and therapeutic approach to adolescent endometriosis, emphasizing the sequential steps and multidisciplinary care involved. Abbreviations: MRI, magnetic resonance imaging; NSAIDs, non-steroidal anti-inflammatory drugs.

Endometriosis is an estrogen-dependent chronic inflammatory condition of unclear etiology, characterized by the presence of endometrium-like epithelium and/or stroma outside the uterus [1]. Primarily affecting women of reproductive age, it can begin as early as the onset of menarche during adolescence and may persist beyond menopause [2,3]. Although its exact prevalence remains unknown, it is estimated that approximately 10% of women and girls of reproductive age are affected, representing roughly 190 million individuals worldwide [4]. Around 67% of endometriosis patients report experiencing symptoms before the age of 20 [5]. The diagnostic and therapeutic pathway for adolescent endometriosis involves several critical steps, from symptom recognition to multidisciplinary follow-up (Figure 1). The most common symptom of endometriosis during adolescence is severe menstrual pain, which interferes with daily activities, social relationships, and school attendance and is poorly responsive to non-steroidal anti-inflammatory drugs and/or oral contraceptives [6,7,8]. Other common symptoms include chronic pelvic pain (CPP), dyspareunia, dysuria, dysmenorrhea, abnormal menstruation, infertility, and gastrointestinal discomfort [9]. On average, patients spend about 10 years searching for a diagnosis.

During adolescence, the diagnosis of endometriosis is often delayed, and the risk of misdiagnosis is high due to the overlap of symptoms with normal menstruation, the varied presentation in younger patients, and the lack of awareness in the medical community [10,11,12]. The prevalence of endometriosis in adolescence varies depending on the study in the literature, the population being examined, and the diagnostic methods used. However, it remains high and represents a significant issue in this age group (Table 1). A definitive diagnosis can be made through laparoscopy; however, given the well-recognized delays in diagnosis, a clinical diagnosis focusing on history, physical examination, and imaging has proven to be a valuable adjunct to diagnosis [13,14,15]. Adenomyosis, historically considered a disease of multiparous women, is increasingly recognized in adolescents, often in conjunction with endometriosis. It is characterized by the ectopic presence of endometrial glands and stroma within the myometrium, leading to dysmenorrhea, menorrhagia, and chronic pelvic pain—symptoms overlapping with those of endometriosis [16]. Despite the limited availability of pediatric-specific data, studies suggest that adenomyosis exacerbates pain severity in adolescents diagnosed with endometriosis. The preferred diagnostic approach in young patients relies on transvaginal or transabdominal ultrasound (US) and magnetic resonance imaging (MRI). Since adenomyosis often coexists with endometriosis, therapeutic strategies largely overlap. However, data on adenomyosis in adolescents remain limited, as most studies focus on adult women [17]. Deep infiltrating endometriosis (DIE) is a severe form of endometriosis with deep infiltration below the peritoneum, commonly affecting the rectovaginal space, bladder, and uterosacral ligaments. Though more frequent in adults, early-stage DIE can occur in adolescents, making early detection crucial [18]. Symptoms include severe dysmenorrhea, bowel dysfunction, dysuria, and deep dyspareunia. MRI is the preferred imaging tool, offering better detection of deep nodules compared to ultrasound. While there is a strong overlap with endometriosis, DIE is more aggressive and often needs a multidisciplinary approach with targeted surgery in order to ensure long-term symptom control and fertility preservation in adolescents [18]. Several treatment options are available for endometriosis, including both surgical and pharmacological approaches, although a definitive cure is still lacking [19]. Treatment responses vary among individuals, with some patients experiencing persistent or recurring symptoms that are debilitating [20,21]. The recent literature suggests that endometriosis negatively impacts the mental well-being of adult patients, affecting their work environment, family relationships, social life, and self-esteem, and contributing to depression and anxiety [22,23,24,25,26,27].

Adolescence is a critical period for emotional and psychological development, and the onset of a chronic illness such as endometriosis can significantly affect adolescents’ well-being and social life. This narrative review was conducted to synthesize current knowledge on the psychological and clinical implications of endometriosis in adolescence.

## 2. Methods

For this narrative review, we conducted an electronic literature search using MEDLINE/PubMed, Scopus, and Web of Science databases to identify articles on endometriosis in adolescence published between January 1999 and January 2025. The following keywords and Medical Subject Headings (MeSH) terms were used in various combinations to identify relevant studies: “Endometriosis” (unique ID: D004715), “Adolescent” (unique ID: D000293), “Mental health” (unique ID: D008603), “Quality of life” (unique ID: D011788), “Diagnosis” (unique ID: D003933), “Therapy” (unique ID: D013812), and “Ultrasonography” (unique ID: D014463). Eligible studies included original research articles (randomized and non-randomized clinical trials, prospective observational studies, retrospective cohort studies, and case–control studies); review articles focusing on the psychological and clinical aspects of endometriosis in adolescence; and studies evaluating diagnostic and treatment strategies, including psychological interventions. Exclusion criteria were as follows: non-English-language manuscripts; articles lacking detailed information on diagnostic or treatment approaches relevant to endometriosis, in particular for adolescence; and conference abstracts, letters to the editor, and case reports. The literature selection was conducted independently by two authors (F.Pa. and R.P.) who screened and reviewed all the studies meeting the inclusion criteria. A total of 215 articles were included, covering pathogenetic theories, epidemiology, psychological implications, imaging-based diagnosis, and treatment options, providing a comprehensive overview of endometriosis in adolescence.

## 3. Integrated Insights into Endometriosis and Emotional Well-Being

### 3.1. Psychological Impact on Adolescence

Endometriosis symptoms, primarily chronic severe pelvic pain and dysmenorrhea, which are often severe and unpredictable, can significantly affect the daily life and basic functioning of adolescents [35,36]. Severe dysmenorrhea, in particular, has been associated with an increased risk of absenteeism and the need for pain medication [3,37,38,39,40]. Absenteeism rates are higher in adolescent patients who also report gastrointestinal symptoms, such as vomiting and diarrhea during menstruation [40]. Furthermore, dysmenorrhea has been shown to significantly decrease subjective sleep quality, sleep efficiency, and rapid eye movement due to elevated nocturnal body temperatures and higher morning estrogen concentrations [41]. Sleep disturbances further contribute to reduced quality of life in these patients, exacerbating the impact of pain on daytime functioning [42].

CPP and severe dysmenorrhea have been correlated with negative perceptions of general health and psychological distress in adolescents, who also experience high rates of somatization [3,43]. CPP is the primary driver of anxiety and depressive symptoms [44], and the presence of a mental health condition may exacerbate the perception of pain [24,45]. Adolescents with CPP are more likely to experience moderate-to-severe mood disturbances compared to their peers without chronic pain conditions [43,46].

Adolescents with endometriosis face various physical, hormonal, and emotional changes associated with growth. A diagnosis of a chronic, painful condition such as endometriosis can disrupt this critical developmental phase [47,48]. The negative impact of endometriosis on school attendance, academic performance, and social engagement can adversely affect self-esteem and self-confidence, potentially leading to emotional dysregulation and long-lasting psychological effects [2]. This association may be more pronounced in individuals reporting a higher symptom burden [46,49]. Decreased social interaction and withdrawal from activities can be attributed to factors such as pain, bleeding, fatigue, depression, feelings of isolation, body image dissatisfaction, low self-esteem, and lack of self-confidence. These factors correlate with diminished perceived social support and lower self-rated emotional well-being [49].

Increased emotional vulnerability leads to difficulties coping with the illness, as well as feelings of frustration, helplessness, anger, and depression due to adolescents’ inability to understand their condition and its progression [50,51]. Girls with endometriosis often experience intense feelings of worthlessness, guilt, and frustration due to disease-related limitations on daily activities, social functioning, independence, and interpersonal relationships [52,53,54]. Negative emotions may be exacerbated by social stigma and the belief that others, including healthcare professionals, perceive their symptoms as “all in their heads”, normalize their pain as part of being a woman, or attribute it to psychological causes [55,56,57,58,59]. The societal normalization of menstrual pain often leads adolescents to hide their symptoms, feeling misunderstood and unrecognized in their struggle [59].

### 3.2. Interplay of Biological Mechanisms Between Endometriosis and Mental Health

Hormonal factors, immunological dysregulation, and chronic inflammation, influenced by both hereditary and environmental factors, play a central role in the pathogenesis of endometriosis. Patients with endometriosis often exhibit elevated levels of estradiol and progesterone [60]. The increased synthesis of estrogen, combined with progesterone resistance and hyperexpression of estrogen receptors, promotes the proliferation and suppresses apoptosis of endometriotic lesions [61]. This hormonal dysregulation also affects the central nervous system (CNS), particularly influencing the synthesis of brain-derived neurotrophic factor (BDNF) [62], as well as other neurotransmitters such as serotonin, increasing the vulnerability to mood disorders in women with endometriosis.

BDNF plays a critical role in the development, survival, and plasticity of neurons in the CNS. Its concentration increases progressively throughout adolescence [63,64,65,66]. Furthermore, BDNF is involved in the regulation and transmission of nociceptive signals in chronic pain syndromes, contributing to the hyperexcitability of spinal nerves and the persistence of pain [67]. Chronic pain, as previously mentioned, is a primary contributor to the development of anxiety and depression in adolescents with endometriosis [68]. Although BDNF is primarily synthesized in the brain, elevated levels of BDNF have been detected in the plasma, peritoneal fluid, and endometrial tissue of women with endometriosis compared to healthy controls, sustained by the local interaction of inflammatory factors and estrogen [69,70,71,72].

Endometriotic lesions are surrounded by an inflammatory microenvironment, driven by cytokines and chemokines (e.g., tumor necrosis factor (TNF)-α, interleukin (IL)-6, and IL-1β) that promote the growth, invasion, and survival of ectopic endometrial cells. This inflammatory milieu not only contributes to tissue damage but also fosters the persistence and expansion of lesions. The microenvironment, rich in immune cells, cytokines, and extracellular matrix components, supports the local immune response, which may lead to fibrosis, scarring, and the formation of adhesions. In this setting, immune cells such as T cells, B cells, and natural killer cells fail to properly recognize and eliminate ectopic endometrial cells, while macrophages and dendritic cells are overactivated, thereby promoting sustained inflammation. Elevated levels of pro-inflammatory cytokines, including ILs and TNF-α, are linked to increased pelvic pain and other symptoms of endometriosis and may also affect brain function, contributing to the development of mood disturbances [73,74].

In the early stages of the disease, endometriotic cell survival is supported by altered energy metabolism, which is characterized by the activation of aerobic glycolysis pathways [75]. Another significant factor in the pathogenesis of endometriosis is dysbiosis in the gut, vaginal, and uterine microbiomes [76,77,78,79,80,81]. These microbiomes are typically characterized by reduced microbial diversity and an overabundance of Gram-negative bacteria, including genera such as *Shigella* and *Escherichia*, as well as *Prevotella* species [82,83,84]. Protective microbes in the gut, such as *Clostridia*, *Ruminococcus*, and *Lachnospiraceae*, are diminished in women with endometriosis, leading to a reduction in short-chain fatty acids, which are important for maintaining intestinal integrity [85]. Altered epithelial permeability causes bacterial translocation, which serves as a trigger for inflammation, activating both local and systemic immune responses [86].

Pro-inflammatory cytokines such as IL-6, TNF-α, and IL-1β have been linked to depression, anxiety, and decreased quality of life [87,88,89]. These cytokines are particularly elevated in patients with gastrointestinal symptoms such as constipation, bloating, flatulence, vomiting, and nausea [82]. The gut microbiome and vaginal microbiome are interconnected, and microbial dysbiosis in one area can influence microbial balance in the other [73]. Moreover, the microbiome plays a role in modulating pain pathways through the gut–brain axis [90], influencing microglial and astrocytic activity, which leads to increased glutamate levels and decreased gamma-aminobutyric acid levels in central synaptic neurotransmission. This imbalance contributes to pain hypersensitivity and its persistence [91,92].

Finally, the role of the microbiome is bidirectional as it is influenced by mental health and pain perception while also influencing them [93]. Gut inflammation can stimulate the afferent vagal nerve, which in turn affects the hypothalamic–pituitary–adrenal axis, microglial activation, and the kynurenine pathway, inducing symptoms associated with depression such as fatigue, decreased motivation, anhedonia, cognitive impairment, and sleep disruption [94].

### 3.3. Non-Invasive Diagnostic Biomarkers and Digital Tools for Monitoring and Support

Advancements in non-invasive diagnostic biomarkers and digital health technologies are transforming the care of adolescents with endometriosis. The traditional reliance on invasive procedures such as laparoscopy has delayed diagnosis for many, highlighting the urgent need for alternative methods [95,96]. US is the first-line imaging modality for diagnosing adolescent endometriosis, particularly in detecting ovarian endometriomas. Transvaginal US is widely used in adults [97], but in adolescents, transabdominal or transrectal US may be preferable for virgin patients. US can detect endometriomas with high specificity but has limited sensitivity for detecting DIE [31]. MRI is a valuable adjunct in evaluating deep endometriotic lesions, particularly in cases of rectovaginal and bladder involvement. High-resolution imaging is essential for pre-surgical assessment, guiding minimally invasive approaches to avoid excessive ovarian tissue loss [98]. Thus, a multimodal imaging approach—US as a first step, followed by MRI if needed—is recommended to improve early diagnosis and treatment planning in adolescent patients. Non-invasive biomarkers are emerging as promising tools, particularly blood-based markers such as Cancer Antigen 125, which, while widely studied, have limited specificity in early or mild cases [99]. More promising are microRNAs (miRNAs), small non-coding RNA molecules that regulate gene expression and remain stable in blood samples. Certain miRNAs, such as the miR-200 family, show significant potential for early diagnosis, offering a non-invasive alternative for identifying endometriosis [100,101]. Proteomic and metabolomic analyses are further advancing this field, identifying inflammatory markers such as IL-6 and TNF-α [102] as well as altered metabolic profiles in endometriosis patients [103,104,105]. These biomarkers could lead to the development of simple blood or urine tests that detect the condition with greater accuracy. Menstrual fluid also presents an accessible, non-invasive diagnostic medium, as studies have identified unique genetic, epigenetic, and protein signatures in menstrual effluent from patients with endometriosis [106,107]. The application of next-generation sequencing technologies enables comprehensive analysis of these samples, offering an alternative to invasive tissue biopsy [108]. Additionally, exosomes and extracellular vesicles, which are released by cells into bodily fluids and carry proteins, lipids, and nucleic acids, are being studied for their diagnostic potential in endometriosis. These small vesicles contain distinct molecular signatures that could serve as reliable biomarkers for the disease [109,110]. Combining multiple biomarkers into diagnostic panels, such as integrating miRNAs, cytokines, and metabolic profiles, may improve the sensitivity and specificity of non-invasive testing, particularly for adolescents (Table 2).

Alongside biomarker development, digital tools are reshaping how endometriosis is monitored and managed, addressing both the physical and psychological challenges of the disease [139]. Mobile health applications allow adolescents to track symptoms such as pain, menstrual patterns, and emotional well-being. These apps provide real-time data to clinicians, enabling more personalized treatment adjustments, while also offering educational resources to improve disease literacy and reduce stigma [140,141]. Wearable devices such as smartwatches and fitness trackers enhance this capability by monitoring physiological parameters such as sleep quality, stress levels, and activity patterns. For instance, heart rate variability data collected by these devices can provide indirect insights into pain severity and emotional distress, supporting targeted interventions [142]. Telemedicine has emerged as a critical tool for improving access to care, particularly for adolescents who may face barriers such as geographic isolation or discomfort discussing symptoms in traditional settings. Virtual consultations enable regular follow-ups and facilitate multidisciplinary collaboration among healthcare providers [143,144]. Digital communities and online support groups further extend this support by connecting adolescents with peers who share similar experiences, reducing feelings of isolation and providing emotional validation [145,146].

Artificial intelligence (AI) is increasingly being integrated into endometriosis care, with machine learning algorithms analyzing symptom patterns, imaging data, and biomarker profiles to predict disease likelihood. AI-powered chatbots provide adolescents with real-time guidance on managing symptoms and understanding treatment options [147,148]. Virtual reality (VR) offers another innovative avenue for care, particularly in pain management. Immersive VR experiences distract patients from chronic pain, reduce stress, and promote relaxation. Preliminary studies suggest that VR interventions can improve both physical and psychological outcomes, making them a valuable complement to traditional treatments [149]. The integration of these digital tools with emerging biomarkers has the potential to transform clinical practice. Collaborative efforts between researchers, clinicians, and technology developers are essential to validate and optimize these advancements, ensuring that they are accessible to all patients. By combining these innovations with traditional medical and psychological interventions, healthcare providers can offer more personalized, effective, and holistic care, addressing the unique challenges of adolescents with endometriosis.

### 3.4. Psychological and Mind–Body Interventions

Medical treatments for endometriosis typically involve non-steroidal anti-inflammatory drugs for pain management and hormonal therapies aimed at reducing estrogen levels and controlling menstrual cycles. Surgery for ovarian endometriosis should be carefully considered in adolescents; conservative surgical techniques with long-term hormonal suppression provide the best symptom control while preserving fertility (Table 3). 

While these treatments can alleviate physical symptoms, their impact on mental health remains less clear, especially in adolescents. Some studies suggest that hormonal therapies may help reduce depressive and anxiety symptoms by alleviating pain [45], whereas others highlight potential side effects, such as mood swings, fatigue, and decreased libido, which could negatively affect psychological well-being [179]. Furthermore, while hormonal treatments help control symptoms [179], they may not directly address the emotional and psychological challenges of living with a chronic condition such as endometriosis [45].

The timely diagnosis of endometriosis in adolescents is paramount in preventing long-term psychological and physical sequelae. Delays in diagnosis—averaging 7–10 years—are often attributed to the normalization of menstrual pain, inadequate awareness among healthcare providers, and the challenges in differentiating endometriosis from other pelvic pain conditions in younger patients [180,181]. Prolonged diagnostic timelines increase the likelihood of disease progression, worsening symptoms, and the accumulation of negative psychological effects such as chronic anxiety, social withdrawal, and depressive episodes [182,183]. Early recognition and diagnosis allow for prompt intervention, mitigating pain and preventing the cascading effects of chronic illness on adolescents’ education, social development, and emotional health [184,185].

Personalized care models, integrating both medical and psychological treatments, emerge as essential strategies for managing endometriosis in adolescence. Tailoring hormonal therapies, such as combined oral contraceptives or progestins, to the patient’s symptomatology and medical history is critical for optimizing symptom control while minimizing side effects that may exacerbate mood disturbances [152,186]. Additionally, the integration of psychological therapies, such as cognitive behavioral therapy (CBT), addresses the psychosocial burden of the disease by improving pain management, coping skills, and emotional resilience [187,188]. Adolescents diagnosed early are also better candidates for educational interventions, allowing healthcare providers to enhance their understanding of endometriosis and empower them to advocate for their care [181].

Moreover, multidisciplinary care involving pediatricians, gynecologists, psychologists, and pain specialists is increasingly recognized as a gold standard for improving outcomes. This team-based approach facilitates comprehensive management, combining physical treatments with emotional and social support to address the multifaceted nature of endometriosis [181,183].

Future research should focus on refining diagnostic algorithms and exploring innovative non-invasive diagnostic biomarkers to expedite diagnosis in younger populations [189,190]. By identifying adolescents at high risk of endometriosis early and implementing individualized, holistic treatment plans, the long-term mental and physical impact of the disease can be significantly reduced, improving quality of life and psychological well-being [184,191].

Given the strong association between endometriosis and mental health issues, early psychological and mind–body interventions have demonstrated effectiveness in reducing both pain and symptoms of anxiety and depression in adolescents [192]. Specifically, CBT is particularly effective in addressing both the psychological and physical symptoms of endometriosis, helping patients manage pain and improve emotional well-being [193,194].

CBT, which focuses on identifying and changing negative thought patterns, helps adolescents cope with the emotional distress associated with chronic pain and illness [193,194]. Mindfulness-based interventions, including relaxation techniques and mindfulness meditation, have been shown to improve emotional regulation and mental health and reduce pain perception, anxiety, and depression symptoms [195,196].

Yoga has also been highlighted as a promising intervention for adolescents with endometriosis. A study conducted by Gonçalves et al. demonstrated that yoga could improve sleep quality and reduce pain intensity, offering a non-pharmacological complement to traditional treatments [197]. A wide range of studies examined the impact of dietary interventions on managing endometriosis symptoms, consistently suggesting that dietary changes can be beneficial in alleviating pain and other related symptoms (Table 4). Interventions commonly explored include antioxidant-rich diets, vitamin D supplementation, gluten-free diets, and omega-3/6 fatty acids, as well as specific dietary approaches such as low-FODMAP and low-nickel diets [198,199].

While most studies report significant reductions in pain and symptom severity, the generalizability of these findings may be limited due to factors such as high heterogeneity, small sample sizes, and varying methodologies. Overall, the growing body of literature supports the potential of dietary modifications as a complementary strategy in managing endometriosis symptoms, although further research is needed to confirm optimal dietary patterns and interventions.

Finally, educating family members and the broader community about the psychological impact of endometriosis and promoting open communication is essential in reducing feelings of isolation and stigma. The chronic nature of the disease, combined with its often invisible symptoms, can lead to social withdrawal and a lack of understanding from family members and peers [43]. Peer support groups, whether in person or online, can offer adolescents a sense of community and validation, helping them cope with the emotional challenges associated with the disease [200].

These groups foster connections with others who understand their experiences, which is particularly beneficial for reducing the social and psychological burden of the disease. Furthermore, promoting mental health awareness within the community and among healthcare providers can ensure that adolescents receive comprehensive care that addresses both physical and emotional needs [201].

**Table 4:** Dietary interventions and their effects on endometriosis symptoms.

Category	Diet/Nutritional Factor	Key Components	Reported Effects on Endometriosis
**Specific diets**	Mediterranean diet (Ott et al., 2012 [202])	Rich in olive oil, fish, nuts, legumes, fruits, and vegetables	Linked to lower endometriosis risk, reduced pelvic pain, and inflammation
	Low-FODMAP diet (Moore et al., 2017 [203]; van Haaps et al., 2023 [204])	Excludes fermentable oligosaccharides, disaccharides, monosaccharides, and polyols (FODMAPs)	Reduces gastrointestinal and abdominal symptoms common in endometriosis (especially if associated with irritable bowel syndrome)
	Low-nickel diet (Borghini et al., 2020 [205])	Excludes foods that contain a high amount of nickel	May be recommended to reduce gastrointestinal, extra-intestinal, and gynecological symptoms (especially if nickel allergic contact mucositis is present)
	Gluten-free diet (Marziali et al., 2012 [206])	Excludes any foods that contain gluten	May reduce pain in gluten-sensitive individuals with endometriosis
**Specific nutrients**	Omega-3 fatty acids (Nodler et al., 2020 [207])	Found in fatty fish, flaxseed, and walnuts	Reduces inflammation, pain, and prostaglandin production
	Antioxidants (Mier-Cabrera et al., 2009 [208]; Santanam et al., 2013 [209])	Vitamins C and E, selenium, and zinc	Mitigates oxidative stress, reduces pain, and enhances symptom relief
	Vitamin D (Nodler et al., 2020 [207]; Qiu et al., 2020 [210])	Found in fortified foods, sunlight exposure, and fatty fish	Regulates immune function. There is a negative relationship between vitamin D levels and the severity of endometriosis
**Food groups**	Dairy products (Harris et al., 2013 [211]; Nodler et al., 2020 [212]; Qi et al., 2021 [213])	Milk, yogurt, and cheese	An optimal intake of total dairy may be associated with a decreased risk of endometriosis
	Red meat (Parazzini et al., 2004 [214]; Yamamoto et al., 2018 [215])	High intake of processed and red meats	Associated with a higher risk of developing endometriosis and worsened symptoms
	Soy products (Tsuchiya et al., 2007 [216])	Isoflavones (phytoestrogens) found in soy milk and tofu	Higher urinary levels of isoflavones may be associated with a reduced risk of advanced but not minimal–mild-stage endometriosis
	Dietary fibers (Parazzini et al., 2004 [214]; Harris et al., 2018 [217])	Fruits and vegetables	Reduction in the risk of endometriosis with higher consumption of (not all, some) fruits and vegetables

## 4. Conclusions

This narrative review provides a comprehensive and multidisciplinary perspective on adolescent endometriosis, integrating clinical, psychological, and emerging biomarker-based insights. One of its major strengths lies in the broad scope of the discussion, which encompasses not only traditional medical and surgical management but also the psychosocial impact of the disease. By addressing the interplay between chronic pain, hormonal dysregulation, and mental health, this review highlights the need for a holistic, patient-centered approach to treatment. Furthermore, the inclusion of non-invasive diagnostic strategies, digital health tools, and psychological interventions offers an innovative outlook on emerging trends in the management of adolescent endometriosis. This broad perspective is particularly valuable given the complex nature of the disease and the frequent diagnostic delays experienced by young patients.

However, as a narrative review, this study does not include a quantitative meta-analysis of treatment outcomes, which could have provided statistical comparisons of therapeutic efficacy. The heterogeneity of included studies—varying in population size, diagnostic criteria, and treatment approaches—may introduce bias and limit the generalizability of findings. Additionally, much of the available literature on endometriosis treatment is derived from adult populations, making it challenging to draw definitive conclusions about the safety and efficacy of specific interventions in adolescents. Longitudinal studies focused on adolescent-specific outcomes are needed to further clarify the long-term effects of hormonal therapies and the impact of psychological interventions on disease progression and quality of life.

Despite these limitations, this review underscores the importance of early diagnosis, personalized treatment, and a multidisciplinary care model. By synthesizing current knowledge and identifying areas for future research, it serves as a valuable resource for clinicians, researchers, and policymakers striving to improve outcomes for adolescents with endometriosis.

## Figures and Tables

**Table 1:** Prevalence of endometriosis in adolescents across different articles.

Study	Population	Diagnostic Method	Prevalence of Endometriosis
**Zannoni et al., 2024 [16]**	Young women (*n* = 100, 14–24 years) with chronic pelvic pain (CPP)	Ultrasound (US)	25% (US-diagnosed endometriosis); higher prevalence in young women (20–24 years) compared to adolescents (14–19 years)
**Millischer et al., 2023 [28]**	Adolescents (12–20 years) with severe dysmenorrhea	Magnetic resonance imaging	39.3% (121/345) overall; 20.7% (25/121) with ovarian endometrioma (OMA), 88.4% (107/121) with deep infiltrating endometriosis (DIE)
**Martire et al., 2023 [29]**	Young women (12–25 years) with severe dysmenorrhea	US	35.3% (131/371) overall; 41.2% (54/131) with OMA, 16.8% (22/131) with isolated endometrioma, and 53.4% (70/131) with posterior DIE
**Hirsch et al.,** **2020 [30]**	Adolescents with CPP undergoing laparoscopy	Laparoscopy	64% (648/1011; range 25–100%)
**Martire et al., 2020 [31]**	Adolescents (12–20 years) referred for US	US	At least one US feature of endometriosis in 13.3% (36/270); higher detection in adolescents with dysmenorrhea (21%) and dyspareunia (33%)
**Yeung et al.,** **2017 [32]**	Adolescents with laparoscopically proven endometriosis	Laparoscopy	Histologically confirmed in 39% of cases (448/1148) [Global prevalence: up to 80% of adolescents with CPP who fail to respond to medical treatment]
**Janssen et al., 2013 [33]**	Adolescents with CPP	Laparoscopy	62% (543/880; range 25–100%)
**Opoku-Anane and Laufer, 2012 [34]**	Young women (12–21 years) with CPP unresponsive to non-steroidal anti-inflammatory drugs and oral contraceptives	Laparoscopy	98% (115/117); all patients had either stage I or II endometriosis

**Table 2:** Non-invasive diagnostic biomarkers for endometriosis.

Biomarker Type	Sample Type	Diagnostic Potential	Limitations	References
**CA125** **(Cancer Antigen 125)**	Serum	Useful for detecting advanced disease in some cases	Low specificity for early/mild cases	Agic et al., 2008 [111]; Mihalyi et al., 2010 [112]; Socolov et al., 2010 [113]; Vodolazkaia et al., 2012 [114]; Ozhan et al., 2014 [115]; Choi et al., 2019 [116]
**MicroRNAs** **(e.g., miR-200 family)**	Serum	Early diagnostic potential, stable in blood, non-invasive	Need for further validation and standardization	Teague et al., 2010 [117]; Jia et al., 2013 [118]; Suryawanshi et al., 2013 [119]; Wang et al., 2013 [120]; Cosar et al., 2016 [121]; Nisenblat et al., 2019 [122]; Vanhie et al., 2019 [123];
**Cytokines** **(IL-6, TNF-α)**	Serum	Can indicate inflammation and the presence of endometriosis	Limited accuracy in differentiating from other diseases	Xavier et al., 2006 [124]; Othman et al., 2008 [125]; Seeber et al., 2008 [126]; Socolov et al., 2010 [113]; Choi et al., 2019 [116]
**Exosomes/extracellular vesicles**	Blood, urine, menstrual fluid	Contains proteins, lipids, and RNA markers for diagnosis	Still in the early stages of research	Li et al., 2020 [127]; Zhang et al., 2020 [128]; Shan et al., 2022 [129]
**Menstrual fluid** **proteins and genes**	Menstrual fluid	Non-invasive, specific to endometriosis	Requires more validation and standardized protocols	Ji et al., 2023 [130]; Amanda et al., 2024 [131]; Starodubtseva et al., 2024 [132]
**Vascular Endothelial Growth Factor (VEGF)**	Serum, urine	Potential biomarker for early detection and staging	Limited sensitivity in early stages, affected by other conditions	Potlog-Nahari et al., 2004 [133]; Xavier et al., 2006 [124]; Vodolazkaia et al., 2012 [114]
**Long non-coding RNAs**	Serum	Emerging role in the diagnosis and prognosis of endometriosis	Limited standardization and variability in findings	Wang et al., 2016 [134]; Qiu et al., 2019 [135]
**Autoantibodies**	Serum	Can help in the detection of autoimmune responses associated with endometriosis	Lack of specificity for endometriosis	Nabeta et al., 2009 [136]; Gajbhiye et al., 2012 [137]
**Apoptotic markers (e.g., caspase-3)**	Serum	Provides insight into endometrial tissue damage and disease progression	Not widely validated for clinical use	Kaya et al., 2018 [138]

**Table 3:** Medical and surgical management of symptomatic endometriosis.

Treatment	Indication	Mechanism of Action	Key Findings
**Non-steroidal anti-inflammatory drugs**	For pain relief in all stages of treatment (Brown et al., 2017 [150])	Temporarily suppress cyclooxygenase (COX)-1 and COX-2 activity, leading to a reduction in prostaglandin synthesis	Reduces pain effectively without long-term narcotic use [11,151]
**Combined hormonal contraceptives (CHC) (oral, vaginal ring, or transdermal)**	First-line treatment to reduce endometriosis-associated dyspareunia, dysmenorrhea, and non-menstrual pain (Brown et al., 2018 [152]; Jensen et al., 2018 [153]; Grandi et al., 2019 [154])	Suppress follicle-stimulating hormone (FSH) and luteinizing hormone (LH), curbing cell growth and promoting endometrial cell death	Effective in reducing symptoms and suppressing endometriosis [11,151]
**Progestin-only agents (e.g., dienogest)**	First-line treatment alternative to CHC, effective in reducing symptoms (Brown et al., 2012 [155]; Petraglia et al., 2012 [156]; Andres et al., 2015 [157])	Lower FSH and LH levels, inducing shrinkage or regression of endometrial tissue	Reduces pain and promotes endometrial suppression [11,151]
**Levonorgestrel-releasing intrauterine system**	Alternative to CHC, provides localized therapy for symptom management (Lan et al., 2013 [158]; Yoost et al., 2013 [159]; Margatho et al., 2020 [160])	Similar to progestin-only agents, with minimal systemic hormone exposure	Effective for long-term symptom relief, reduces pain [159]
**Gonadotropin-releasing hormone (GnRH) agonists**	For refractory cases, often used with add-back therapy (Brown et al., 2010 [161]; Tang et al., 2017 [162]; Veth et al., 2023 [163])	Prolonged use suppresses steroid hormone production by lowering LH and FSH levels, although initially causes a hormone surge	Need add-back therapy (combination of low-dose hormones) to prevent bone loss and hypoestrogenic symptoms [164,165]
**GnRH antagonists**	A second-line treatment (e.g., if CHC or progestogens have been ineffective) owing to their side effect profile (Taylor et al., 2017 [166]; Donnez et al., 2020 [167]; Osuga et al., 2021 [168])	Competitively binds to GnRH receptors in the pituitary gland, leading to immediate suppression of LH and FSH levels without the initial hormone flare seen with GnRH agonists	Similar to GnRH agonists [169]
**Aromatase inhibitors**	For severe cases resistant to other treatments (Ferrero et al., 2011 [170]; Almassinokiani et al., 2014 [171])	Prevents the transformation of androgens into estrogens, diminishing endometrial cell proliferation	Effective in combination with CHC, progestins, and GnRH agonists or antagonists [172]
**Androgens (e.g., danazol)**	Second-line treatment for refractory cases or patients with contraindications to other treatments (Ferrero and Barra, 2022 [173])	Act as antiestrogens by inhibiting enzymes responsible for steroid production and reducing gonadotropin secretion	Reduces endometriosis symptoms, but typically avoided in adolescents and young women due to long-term androgenic effects [174]
**Selective progesterone receptor modulators**	Emerging therapy for pain and lesion reduction (Fu et al., 2017 [175])	Antagonistic effects on progesterone receptors in the endometrium, leading to the suppression of endometrial proliferation and induction of atrophy, contributing to symptom relief	Reduces endometrial growth with fewer side effects compared to GnRH agonists; long-term safety data are limited, particularly regarding potential endometrial effects, such as changes in endometrial histology [176]
**Surgical management**	For refractory cases with severe pain, organ dysfunction, or infertility risk (Tyson et al., 2024 [177])	Removes endometriotic lesions to relieve symptoms and prevent recurrence	Effective in selected cases but requires specialized surgical expertise; postoperative hormonal therapy reduces recurrence [178]

## Data Availability

Not applicable.
